# Retrospective study of alveolar ridge preservation compared with no alveolar ridge preservation in periodontally compromised extraction sockets

**DOI:** 10.1186/s40729-021-00305-2

**Published:** 2021-03-26

**Authors:** Jungwon Lee, Junseob Yun, Jung-Ju Kim, Ki-Tae Koo, Yang-Jo Seol, Yong-Moo Lee

**Affiliations:** 1 One-Stop Specialty Center, Seoul National University Dental Hospital, Seoul, Republic of Korea; 2 Department of Periodontology, School of Dentistry and Dental Research Institute, Seoul National University, Seoul, Republic of Korea

**Keywords:** Bone substitutes, Guided tissue regeneration, Bone regeneration, Clinical research, Clinical trials

## Abstract

**Background:**

To minimize alveolar bone resorption, alveolar ridge preservation (ARP) has been proposed. Recently, interest in improving the feasibility of implant placement has gradually increased, especially in situations of infection such as periodontal and/or endodontic lesions. The aim of this study was to investigate if ARP improves feasibility of implant placement compared with no ARP in periodontally compromised sites. Secondary endpoints were the necessity of bone graft at the time of implant placement and implant failure before loading at ARP compared with no ARP.

**Material and methods:**

This retrospective study was performed using dental records and radiographs obtained from patients who underwent tooth extraction due to chronic periodontal pathology. Outcomes including the feasibility of implant placement, horizontal bone augmentation, vertical bone augmentation, sinus floor elevation, total bone augmentation at the time of implant placement, and implant failure before loading were investigated. Multivariable logistic regression analysis was performed to examine the influence of multiple variables on the clinical outcomes.

**Results:**

In total, 418 extraction sites (171 without ARP and 247 with ARP) in 287 patients were included in this study. The ARP group (0.8%) shows significantly lower implant placement infeasibility than the no ARP group (4.7%). Horizontal and vertical bone augmentations were significantly influenced by location and no ARP. Total bone augmentation was significantly influenced by sex, location, and no ARP.

**Conclusion:**

ARP in periodontally compromised sites may improve the feasibility of implant placement. In addition, ARP attenuate the severity of the bone augmentation procedure.

**Supplementary Information:**

The online version contains supplementary material available at 10.1186/s40729-021-00305-2.

## Background

During the healing period following tooth extraction, the alveolar ridge undergoes horizontal and vertical resorption [1]. Biological changes take place rapidly in the first 3–6 months after tooth extraction, and bone resorption persists throughout the person’s lifetime at a slow pace [2]. This is of important concern at the time of implant placement because insufficient bone quantity may compromise the feasibility of implant placement, increase the necessity of bone augmentation, and increase the likelihood of implant failure [3–5].

To minimize alveolar bone resorption, alveolar ridge preservation (ARP), defined as immediate guided bone regeneration (GBR) following tooth extraction, has been proposed [6–8]. Although ARP cannot preserve the alveolar bone width and height completely, unlike what the term suggests, there is evidence that the intervention minimizes alveolar bone shrinkage after tooth extraction, which results in a favorable environment for implant placement [9].

Recently, interest in improving the feasibility of implant placement has gradually increased, especially in situations of infection such as periodontal and/or endodontic lesions. In clinical situations, the most frequently extracted teeth are those with periodontal and/or endodontic lesions [3, 10]. Bone destruction resulting from periodontal or endodontic disease can aggravate alveolar bone shrinkage [11]. Another study demonstrated that an infected socket could induce erratic healing, which is an incomplete bone healing pattern present after more than 6 months following tooth extraction, unexpectedly increasing the entire treatment period [3].

To avoid marked bone resorption following tooth extraction in infected sites, ARP with secondary intention healing has been proposed [12, 13]. Our recent study revealed that ARP in periodontally compromised sockets with thorough debridement and systemic antibiotics can be a safe procedure, showing 2.7% of infection rate and 0.7% of reinfection rate that need removal of implanted biomaterials [14].

The aim of this retrospective study was to investigate whether ARP improves feasibility of implant placement compared with no ARP in periodontally compromised sites. Secondary endpoints were the necessity of bone graft at the time of implant placement and implant failure before loading at ARP compared with no ARP.

## Materials and methods

This retrospective study was performed according to the STROBE guidelines [15].

### Study design

**Fig. 1:**
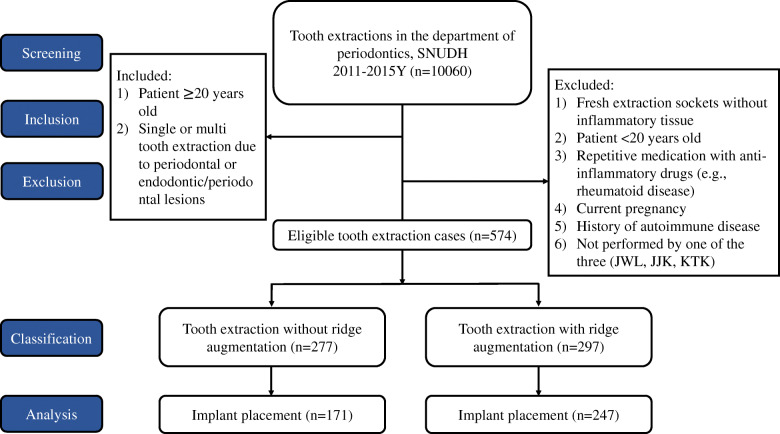
Flowchart of this study. A total of 10,060 patients subjected to tooth extractions at the Department of Periodontology, Seoul National University Dental Hospital were identified throughout chart review. A total of 574 extraction sites were eligible for this study that met inclusion criteria. The number of extraction sites without ARP was 277, and the number of extraction sites with ARP was 297. Finally, 171 of the 277 extraction sites in the no ARP group and 247 of the 297 extraction sites in the ARP group received implant placement

This retrospective study was approved by the Institutional Review Board (IRB) at Seoul National University (IRB073/06-18) and followed the Declaration of Helsinki. Waiver of informed consent was approved according to IRB guidelines for chart review of anonymous patient data. A chart review including all patients who had tooth extraction due to periodontal or endodontic/periodontal lesions at Seoul National University Dental Hospital (SNUDH) from 2011 to 2015 was performed by three periodontists (JWL, JJK, and KTK). An additional chart review was performed to identify patients who underwent implant placement following tooth extraction at Seoul National University Dental Hospital (SNUDH) from 2011 to 2018 that was performed by one of the three periodontists (JWL, JJK, and KTK). All treatment processes at dental hospitals were recorded in the electronic health record (Electronic Medical Record System ver. A16.0628.1035. DSGR.SYBASE. FOLDER, ELSA) or physical dental charts retained at the hospital. All chart records of these patients’ dental records were audited to categorize two treatment modalities: implant placement following ARP and implant placement with no ARP. Due to the lack of cone beam computed tomography data at the time of tooth extraction, the configuration of bone destruction which surrounded the periodontally compromised teeth could not be figured out. The bone graft procedure performed immediately after tooth extraction was defined as ARP. Before tooth extraction, all of the patients were explained and asked to receive ARP. The procedure was performed only to patients who agreed to receive ARP (Fig. 1).

The ARP group in the present study was collected from our previous study [14]. The inclusion criteria have been previously reported in detail. In brief, the patients in the ARP group were treated with ARP at the extraction sockets with periodontal pathosis using a resorbable membrane without flap elevation.

### Case definition of periodontally compromised extraction sockets

The definition of periodontally compromised extraction sockets was provided in detail in a previous study [14]. Periodontally compromised extraction sockets were defined as sites where teeth had been removed due to chronic periodontal or combined endodontic/periodontal lesions. Periodontal indication was defined as teeth with mobility > 2°, a probing depth (the deepest site) greater than 8 mm, and marginal bone loss that narrowed apically. Combined endodontic–periodontal indication was defined as teeth displaying a radiographic periapical lesion greater than 4-mm diameter and exhibiting a negative response to the electric pulp test with periodontal characteristics as mentioned above.

### Subject selection and criteria

Some tooth extraction sites did not receive an implant restoration when the patients received missing tooth with fixed partial denture or implant restoration in other clinic. Those cases were excluded in this study. An electronic record and chart of these patients were inspected to identify ARP procedures conducted before implant placement.

### Data extraction

Two calibrated reviewers (JL and JJK) identified the cases meeting the criteria for periodontally compromised extraction sockets. Through electronic record and chart in Seoul National University Dental Hospital, self-reported patient demographics, including age, sex, hypertension (yes/no), diabetes mellitus (yes/no), tuberculosis (yes/no), hepatitis (yes/no), osteoporosis (yes/no), smoking (yes/no), implant placement location (maxillary—anterior/posterior, mandibular—anterior/posterior), reason for extraction, ARP details (date, graft material, membrane use, infection, reinfection), surgeon (resident/specialist), bone quality [16] (Type 1, Type 2, Type 3, and Type 4), feasibility of implant placement at the planned surgery date (yes/no), bone augmentation at implant surgery (horizontal, vertical, sinus floor elevation, and total), implant details (connection type, length, diameter, and system), implant surgery protocol (1-stage/2-stage), and periods from extraction to implant placement (< 3 months, 3 to 6 months, > 6 months) before loading, were collected. Implant removal before loading for any reason was regarded as an implant failure, and all other implants that were not removed were regarded as surviving implants.

### Statistical analysis

All clinical data were de-identified and stored in a Microsoft Excel spreadsheet (Microsoft Corp, Redmond WA, USA). Statistical analyses were performed using IBM SPSS Statistics for Windows, version 22.0 (IBM Corp, Armonk, NY, USA). Multivariable logistic regression analysis was used to investigate the influence of multiple variables on the feasibility of implant placement, bone augmentation at implant surgery (horizontal bone augmentation, vertical bone augmentation, sinus floor elevation, and total bone augmentation), and implant failure before loading. In the case of sinus floor elevation, only cases at the maxillary posterior region were used. *P* values < 0.05 were regarded as statistically significant.

## Results

A total of 10,060 patients subjected to tooth extractions at the Department of Periodontology, SNUDH, from 2011 to 2015 were identified by chart review (Fig. 1). The tooth extraction cases that met the inclusion criteria were included, and 574 tooth extraction sites were eligible. The number of tooth extractions sites without ARP was 277, and there were 297 tooth extraction sites with ARP. Finally, 171 of 277 extraction sites in the no ARP group underwent implant placement, while 247 of 297 sites in the ARP group underwent implant placement several months later. The characteristics of the patients and surgical sites included in this study are presented in Table 1. The bone graft materials used in ARP were deproteinized bovine bone mineral (Bio Oss®; Geistlich Pharma, Wolhusen, Switzerland), hydroxyapatite and β-tricalcium phosphate mixed with collagen type I (OSTEON™ II Collagen; Genoss, Ganggyo, Korea), Ninety percent of deproteinized bovine bone mineral and 10% porcine collagen (Bio Oss® Collagen; Geistlich Pharma, Wolhusen, Switzerland), bovine-origin bone substitute material (OCS- B®; NIBEC, Seoul, Korea), or freeze-dried bone allografts (SureOss™; HansBiomed, Seoul, Korea). The upper area of extraction socket was covered with resorbable collagen membrane (Bio-Gide®, Geistlich Pharma AG, Wolhusen, Switzerland) or not following the application of bone graft material (Table 1).

**Table 1:** Characteristics of patients and surgical sites included in the study

				No ARP	ARP
General characteristics	Age			58.06 ± 11.79	50.32 ± 10.59
		< 65		130	220
		≥ 65		41	27
	Sex	Male		99	138
		Female		72	109
	Systemic disease	Hypertension	Yes	45	51
			No	126	196
		Diabetes mellitus	Yes	12	26
			No	159	221
		Tuberculosis	Yes	0	2
			No	171	245
		Hepatitis	Yes	5	7
			No	166	240
		Osteoporosis	Yes	9	5
			No	162	242
		Smoking	Yes	25	33
			No	146	214
Site characteristics	Location	Maxillary anterior		17	31
		Maxillary posterior		89	105
		Mandibular anterior		10	10
		Mandibular posterior		55	101
	Reason for extraction	Periodontal		147	161
		Endo-perio		24	86
Ridge augmentation	Bone graft material	A		-	109
		B		-	8
		C		-	117
		D		-	5
		E		-	8
	Membrane	F		-	91
		G		-	11
Surgeon	Resident			136	160
	Specialist			35	87
Total				171	247

A: Bio Oss® (Geistlich Pharma, Wolhusen, Switzerland), B: OSTEON™ II Collagen (Genoss, Ganggyo, Korea). C: Bio Oss® Collagen (Geistlich Pharma, Wolhusen, Switzerland), D: OCS- B® (NIBEC, Seoul, Korea), E: SureOss™ (HansBiomed, Seoul, Korea), F: Biogide (Geistlich Pharma, Wolhusen, Switzerland), G: none

The clinical outcomes are presented in Table 2. Data are presented as the mean ± the standard deviation (SD) for continuous variables and as the frequency for categorical variables.

**Table 2:** Clinical outcomes of the two groups at implant placement

			No ARP	ARP
Bone quality		Type 1	2	4
		Type 2	108	163
		Type 3	59	80
		Type 4	2	0
Feasibility of implant placement		Yes	163	245
		No	8	2
Bone augmentation at implant surgery	Horizontal bone augmentation	Yes	66	45
		No	105	202
	Vertical bone augmentation	Yes	26	3
		No	145	244
	Sinus floor elevation	Yes	27	16
		No	144	231
	Total	Yes	77	58
		No	94	189
Implant	Type	Bone level, internal	144	172
		Bone level, external	23	75
		Tissue level	4	0
	Length	≤ 8.5 mm	10	15
		8.6–12.9 mm	157	230
		≥ 13 mm	4	2
	Diameter	< 3.75 mm	3	18
		3.75 to 4.5 mm	113	126
		> 4.5 mm	55	103
	System	A	2	23
		B	5	8
		C	73	109
		D	20	75
		E	66	12
		F	5	20
Surgical protocol		1-stage	121	155
		2-stage	50	92
Periods (months) from extraction to implant placement		Mean ± SD	4.98 ± 4.07	6.29 ± 3.50
		< 3 months	50	0
		3 to 6 months	121	124
		> 6 months	0	123
Implant failure before loading			1	4

A: Astra (Dentsply Sirona, North Carolina, USA), B: ITI Dental Implant (Institut Straumann AG, Waldenburg, Switzerland), C: USII, and TSIII (Osstem implant, Seoul, Korea), D: Implantium and Superline (Dentium, Seoul, Korea) E: Luna, and Sola (Shinhung, Seoul, Korea) F: EB (Neobiotech, Seoul, Korea)

### Infeasibility of implant placement

Infeasibility of implant placement means the cases that did not perform implant installation due to the lack of achieving primary stability including implant spinning or loosening on the day of implant surgery. Eight (4.7%) patients in the no ARP group exhibited implant placement infeasibility, while two patients (0.8%) in the ARP group presented implant placement infeasibility on the planned surgery day. When the implant was not initially placed due to the lack of bone quantity, bone augmentation was performed, and several months later, implant placement was performed successfully.

Analysis of potential risk factors for the infeasibility of implant placement using a multivariable logistic regression model showed that location (odds ratio [OR] of maxillary anterior = 10.32, 95% confidence interval [CI] = 1.39 to 76.43, *P* = 0.022) and no ARP (OR = 10.28, 95% CI = 1.80 to 58.69, *P* = 0.009) significantly influenced infeasibility of implant placement (Table 3).

**Table 3:** Multivariable logistic regression analysis of hard tissue augmentation at implant placement. *N/A* not available

Variables			Infeasibility of implant placement		Bone augmentation								Implant failure before loading	
					Horizontal		Vertical		Sinus floor elevation^a^		Total^b^			
			OR (95% CI)	*P* value	OR (95% CI)	*P* value	OR (95% CI)	*P* value	OR (95% CI)	*P* value	OR (95% CI)	*P* value	OR (95% CI)	*P* value
Site related	Location	Maxillary anterior	10.32 (1.39–76.43)	0.02	0.19 (0.09–0.39)	< 0.01	0.38 (0.07–1.97)	0.25	-	-	0.20 (0.10–0.42)	< 0.01	N/A^c^	N/A^c^
		Maxillary posterior	1.642 (0.29–9.40)	0.58	1.03 (0.60–1.78)	0.90	0.25 (0.08–0.76)	0.02	-	-	0.53 (0.32–0.89)	0.02	N/A^c^	N/A^c^
		Mandibular anterior	N/A^c^	N/A^c^	0.72 (0.25–2.11)	0.55	N/A^c^	N/A^c^	-	-	0.68 (0.23–1.97)	0.47	N/A^c^	N/A^c^
		Mandibular posterior	1	N/A	1	N/A	1	N/A	-	-	1	N/A	1	N/A
	Reason for extraction	Periodontal	0.69 (0.15–3.24)	0.64	1.05 (0.58–1.89)	0.87	0.97 (0.29–3.27)	0.97	1.76 (0.69–4.51)	0.24	1.05 (0.61–1.83)	0.85	0.89 (0.07–11.45)	0.93
		Endo–perio	1	N/A	1	N/A	1	N/A	1	N/A	1	N/A	1	N/A
	Ridge preservation	No	10.28 (1.80–58.69)	0.01	3.68 (2.20–6.14)	< 0.01	15.80 (4.46–56.03)	< 0.01	1.98 (0.91–4.31)	0.09	3.10 (1.93–5.00)	< 0.01	0.28 (0.03–3.14)	0.28
		Yes	1	N/A	1	N/A	1	N/A	1	N/A	1	N/A	1	N/A
Surgeon related		Resident	0.97 (0.18–5.26)	0.97	1.32 (0.77–2.28)	0.32	1.50 (0.54–4.16)	0.44	0.51 (0.20–1.28)	0.15	1.09 (0.65–1.82)	0.75	0.59 (0.07–4.93)	0.63
		Specialist	1	N/A	1	N/A	1	N/A	1	N/A	1	N/A	1	N/A

^a^Logistic regression analysis was performed in only maxillary posterior region.

^b^Total GBR means all types of hard tissue augmentation, including horizontal GBR, vertical GBR, or sinus floor elevation.

^c^Estimates are not reliable because there were too few observations.

### Bone augmentation at implant surgery

Horizontal bone augmentation was carried out when bone width was deficient, resulting in the implant fixture exposure with bone dehiscence or fenestration. It was performed in sixty-six (38.6%) patients in the no ARP group and forty-five patients (18.2%) in the ARP group (Table 2). Vertical bone augmentation was performed when bone height was deficient, and the implant fixture was exposed with no bony walls. It was conducted in twenty-six patients (15.2%) in the no ARP group and in three patients (1.2%) in the APR group (Table 2). Sinus floor elevation was performed in twenty-seven patients (15.8%) in the no ARP group and in sixteen patients (6.5%) in the ARP group (Table 2). Total bone augmentation occurred in seventy-seven patients (45.0%) in the no ARP group and in fifty-eight patients (23.5%) in the ARP group (Table 2).

Analysis of potential risk factors in horizontal bone augmentation using a multivariable logistic regression model showed that location (OR of maxillary anterior = 0.19, 95% confidence interval [CI] = 0.09 to 0.39, *P* < 0.01) and no ARP (OR = 3.68, 95% CI = 2.20 to 6.14, *P* < 0.01) significantly influenced horizontal bone augmentation (Table 3).

Analysis of potential influencing factors in vertical bone augmentation using a multivariable logistic regression model showed that location (OR of maxillary posterior = 0.25, 95% confidence interval [CI] = 0.08 to 0.76, *P* = 0.02) and no ARP (OR = 15.80, 95% CI = 4.46 to 56.03, *P* < 0.01) significantly influenced vertical bone augmentation (Table 3).

Analysis of potential risk factors for sinus floor elevation using a multivariable logistic regression model showed that the impact of no ARP on sinus floor elevation bordered on but was not less than the accepted level of significance (OR = 1.98, 95% CI = 0.91 to 4.31, *P* = 0.09) (Table 3).

Analysis of potential risk factors in total bone augmentation using a multivariable logistic regression model showed that sex (OR = 0.61, 95% CI = 0.37 to 0.99, *P* = 0.04), location (OR of maxillary anterior = 0.20, 95% CI = 0.10 to 0.42, *P* < 0.01; OR of maxillary posterior = 0.53, 95% CI = 0.32 to 0.89, *P* = 0.016), and no ARP (OR = 3.10, 95% CI = 1.93 to 5.00, *P* < 0.01) significantly influenced total bone augmentation (Table 3, Table S1).

### Implant failure before loading

One (0.6%) patient without ARP exhibited implant failure before loading, while four patients with ARP (1.6%) presented implant failure before loading (Table 2). The details of the failed implants are presented in Table 4. All sites where the implants failed before loading were maxillary except for one case.

**Table 4:** Details of failed implants before loading

No.	Site	Age (Y)	Sex	Ridge preservation?	Feasibility of implant placement	Length (mm)	Diameter (mm)	Implant type	Bone quality	Bone augmentation			
										Horizontal	Vertical	Sinus floor elevation	Total
1	14	73	M	No	Yes	8	3.5	Bone level, internal	Type 3	No	No	No	No
2	43	61	M	No	Yes	10	4.0	Bone level, internal	Type 3	Yes	Yes	No	Yes
3	13	57	M	No	Yes	10	4.0	Bone level, internal	Type 3	Yes	No	No	Yes
4	13	53	M	Yes	Yes	10	4.0	Bone level, external	Type 2	No	No	No	No
5	15	57	M	No	Yes	10	5.0	Bone level, internal	Type 3	No	No	No	No

Analysis of potential risk factors in implant failure before loading using a multivariable logistic regression model showed no significant results (Table 3, Table S1).

### Clinical outcomes of infection following ARP

There were eight patients exhibiting inflammatory symptoms requesting additional treatment following ARP, as reported in a previous study [14]. All patients except one were treated with implant restoration. There was no failure of implant placement feasibility and no implant failure before loading (Table 5). Only one patient who showed reinfection underwent horizontal bone augmentation at the time of implant placement.

**Table 5:** Clinical outcomes of infected ridge preservation sites

No.	Site	Age (Y)	Sex	Removal of biomaterials due to reinfection	Feasibility of implant placement	Length (mm)	Diameter (mm)	Implant type	Bone quality	Bone augmentation				Implant failure before loading
										Horizontal	Vertical	Sinus floor elevation	Total	
1	47	49	F	No	Yes	8	4.5	Bone level, internal	Type 2	No	No	No	No	No
2	26	48	M	No	Yes	10	5.0	Bone level, internal	Type 3	No	No	No	No	No
3	25	48	M	No	Yes	8	4.0	Bone level, internal	Type 3	No	No	No	No	No
4	25	48	M	Yes	Yes	8	4.0	Bone level, internal	Type 2	Yes	No	No	Yes	No
5	46	64	M	No	Yes	8.5	5.0	Bone level, internal	Type 2	No	No	No	No	No
6	47	56	M	No	Yes	10	5.0	Bone level, internal	Type 2	No	No	No	No	No
7	37	43	F	No	Yes	8.5	5.0	Bone level, external	Type 3	No	No	No	No	No

## Discussion

In the previous study, ridge preservation/augmentation in periodontally compromised extraction site showed high safety rate of 99.3%, and biomaterials were removed in two cases (0.7%). However, if the regenerated bone results in insufficient bone quality or/and quantity to obtain proper primary stability when the implants are placed, the procedure cannot be recommended. The aim of this retrospective study was to investigate the feasibility of implant placement, bone augmentation at implant surgery, and implant failure before loading at ARP sites compared with those without ARP in periodontally compromised sockets. The feasibility of implant placement and bone augmentation was influenced by ARP. But there are confounding factors, which are tooth position, healing time for implant placement, individual healing ability, and bone quality of the patients, the amount of alveolar bone destruction due to periodontitis.

Eight patients (4.7%) in the no ARP group did not undergo implant placement, and bone augmentation was conducted several months following tooth extraction because of poor bone quality or insufficient bone quantity. According to a previous study, it was reported that the proportion of erratic healing without implant placement was 5.41% when spontaneous healing was induced without any intervention in the infected extraction socket, which was similar to the findings presented in our study [3]. However, only 2 patients (0.8%) were observed in the ARP group for whom implant placement was not performed and for whom additional bone augmentation was conducted several months following tooth extraction due to poor bone quality and quantity. When ARP was performed, the feasibility of implant placement seemed to increase; however, the procedure did not always guarantee the feasibility of implant placement. Bone defect types could not be distinguished, and sufficient consideration was not made in this study due to a lack of information about the extraction socket. It is considered that unfavorable bone configuration and destruction size may affect the outcome. Although there is a classification system for extraction socket according to the hard and soft tissue deficiency [17, 18], the feasibility of implant placement following ARP has not been investigated. Therefore, it will be necessary to investigate the bone healing patterns based on configuration and size of extraction socket in the future.

A high odds ratio in implant placement feasibility was observed in maxillary anterior teeth compared with that for mandibular posterior teeth. Considering that the anterior region was lower than the posterior region with respect to total bone augmentation, it is presumed that the reason for the decreased feasibility of implant placement in the anterior region was not due to insufficient bone quantity but due to low bone quality. The previous literature suggests that the maxilla is of poorer bone quality than the bone quality of the mandible [19], especially in the posterior maxilla [20]. Due to the variety of implant designs included in this study, it was difficult to draw a definitive conclusion about the relationship between implant design and implant placement feasibility. Within the limitation, it is recommended to use implant systems having tapered designs and/or aggressive threads or apply drilling techniques such as under-drilling or osseodensification in the bone with poor bone quality in order to increase the primary stability [21–23].

In this study, according to the logistic regression analysis, the potential risk factor for horizontal, vertical, and total bone augmentations was no ARP. Considering that ARP is a procedure for bone augmentation, ARP does not always prevent additional bone augmentation when implant placement is performed [24]. However, it should be noted that vertical bone augmentation can be reduced by performing ARP. Vertical bone augmentation is considered to be a more challenging technique than the technique required to achieve horizontal bone augmentation [25, 26]. By reducing the necessity of vertical bone augmentation, it may be possible to reduce the difficulty of additional bone augmentation.

According to the logistic regression analysis, the maxillary posterior region was another potentially influencing factor in vertical bone augmentation (OR of maxillary posterior = 0.25, 95% confidence interval [CI] = 0.079 to 0.762, *P* = 0.015). In the case of the maxillary posterior region, in addition to the vertical bone augmentation procedure, sinus floor elevation can be performed when the vertical bone dimension is insufficient for implant placement. For this reason, the maxillary posterior region seems to have an offset effect of vertical bone augmentation. In addition, it is thought that sinus floor elevation tends to be more favored than vertical bone augmentation by surgeons.

Analysis of potential risk factors in sinus floor elevation using a multivariable logistic regression model showed that the impact of no ARP on sinus floor elevation was not significant (OR = 1.98, 95% CI = 0.911 to 4.308, *P* = 0.085), which is not in agreement with a recent clinical study demonstrating that ARP may reduce the necessity of sinus floor elevation [27]. Sinus floor elevation is dependent on the residual bone height and implant length planned by the surgeon. To verify that ARP indeed reduces the need for sinus floor elevation by attenuating sinus pneumatization, quantitative radiographic analysis will be needed in the future.

Overall implant failure before loading occurred in 5 patients (1.2%), with one patient (0.6%) in the no ARP group and four patients (1.6%) in the ARP group. Unlike the findings of a previous study, in which an implant survival rate of 100% was reported, some implant failures were found in the present study [28]. This result may be because our study evaluated only periodontally compromised sites, unlike the previous study. In fact, it has been reported that implant failure in sites with periodontitis was higher than that in sites without periodontitis [29]. Although the implant failure rate was not as high in this study as in the previous study, attention should be paid when implant placement is performed in sites with periodontitis history. In addition, late implant failure should be investigated after implant prosthesis delivery to determine whether a comparable bone remodeling process is in place in ARP sites.

A total of eight patients were found to have inflammation in the ARP group, one of whom did not receive an implant restoration. Six patients with inflammation resolved by antibiotics did not undergo further bone augmentation, whereas one patient who experienced reinfection received additional bone augmentation when performing implant placement. Although the procedure is not completely identical, the study related to clinical outcomes following conventional guided bone regeneration and subsequent infection is worth investigating. Clinical outcomes of membrane exposure with infection in conventional GBR showed a comparable amount of bone regeneration compared with that without membrane exposure [30, 31]. As ARP is a hard tissue regeneration procedure, infection following the procedure that cannot be controlled with antibiotics can have a detrimental effect on clinical outcomes.

This study has some limitations inherent to its retrospective design. Several pieces of information, including the detailed surgical protocol, bone graft materials, membranes, and implant type, were heterogeneous. Furthermore, the bone defect size and remaining bone wall could not be classified due to the lack of cone beam computerized tomography before tooth extraction. In addition, because implant survival has only been determined before prosthetic loading, we failed to evaluate the long-term survival and success outcomes of implants at ARP compared to no ARP sites. Well-designed prospective studies are needed to investigate the efficacy of ARP in chronic pathology, and it is of interest if there are certain defect configurations that make ARP more useful in periodontally compromised sites than others.

## Conclusion

Within the limitations of this retrospective chart analysis, the findings herein suggest that ARP may improve the feasibility of implant placement and attenuate the severity of the bone augmentation procedure with similar implant failure before loading compared with sites without ARP that are periodontally compromised.

## Supplementary Information

**Additional file 1: Table S1.** Multivariable logistic regression analysis of hard tissue augmentation at implant placement.

## Data Availability

The datasets generated and analyzed during the current study are available from the corresponding author upon reasonable request.

## Abbreviations

ARP: Alveolar ridge preservation
GBR: Guided bone regeneration
IRB: Institutional Review Board
OR: Odds ratio
CI: Confidence interval
