# Supplementary material for: Retrospective study of alveolar ridge preservation compared with no alveolar ridge preservation in periodontally compromised extraction sockets
Source: Int J Implant Dent. 2021 Mar 26;7:23. doi: 10.1186/s40729-021-00305-2 (PMC7994480; doi:10.1186/s40729-021-00305-2)
Supplement: Supplementary file 1 — Additional file 1: Table S1. Multivariable logistic regression analysis of hard tissue augmentation at implant placement. [file 40729_2021_305_MOESM1_ESM.docx]

Supplement 1. Multivariable logistic regression analysis of hard tissue augmentation at implant placement. N/A: not available

| Variables | | | Infeasibility of implant placement | | Bone augmentation | | | | | | | | Implant failure before loading | |
| --- | --- | --- | --- | --- | --- | --- | --- | --- | --- | --- | --- | --- | --- | --- |
|  |  |  |  |  | Horizontal | | vertical | | Sinus floor elevation^†^ | | total^‡^ | |  |  |
|  |  |  | OR  (95% CI) | P value | OR  (95% CI) | P value | OR  (95% CI) | P value | OR  (95% CI) | P value | OR  (95% CI) | P value | OR  (95% CI) | P value |
| Patient related | Age | <65 | 1 |  | 1 | N/A | 1 | N/A | 1 | N/A | 1 | N/A | 1 | N/A |
|  |  | ≥65 | 0.70  (0.08-6.49) | 0.76 | 1.49  (0.72-3.06) | 0.28 | 1.39  (0.40-4.79) | 0.60 | 2.13  (0.62-7.28) | 0.23 | 1.91  (0.95-3.85) | 0.07 | 3.23  (0.24-44.51) | 0.38 |
|  | Sex | Male | 2.43  (0.54-11.01) | 0.25 | 0.76  (0.45-1.27) | 0.29 | 0.81  (0.31-2.15) | 0.67 | 0.68  (0.29-1.57) | 0.37 | 0.61  (0.37-0.99) | 0.04 | N/A ^§^ | N/A ^§^ |
|  |  | Female | 1 | N/A | 1 | N/A | 1 | N/A | 1 | N/A | 1 | N/A | 1 | N/A |
|  | Systemic disease | Hypertension | N/A ^§^ | N/A ^§^ | 1.12  (0.59-2.12) | 0.74 | 0.51  (0.17-1.51) | 0.22 | 1.05  (0.36-3.00) | 0.94 | 1.23  (0.67-2.25) | 0.51 | 2.92  (0.35-24.28) | 0.32 |
|  |  | Diabetes mellitus | N/A ^§^ | N/A ^§^ | 0.86  (0.35-2.12) | 0.74 | 1.21  (0.19-7.51) | 0.84 | 3.10  (0.35-27.80) | 0.31 | 0.86  (0.36-2.03) | 0.72 | N/A ^§^ | N/A ^§^ |
|  |  | Tuberculosis | N/A ^§^ | N/A ^§^ | N/A ^§^ | N/A ^§^ | N/A ^§^ | N/A ^§^ | N/A ^¶^ | N/A ^¶^ | N/A ^§^ | N/A ^§^ | N/A ^§^ | N/A ^§^ |
|  |  | Hepatitis | N/A ^§^ | N/A ^§^ | 2.16  (0.44-10.56) | 0.34 | N/A ^§^ | N/A ^§^ | N/A ^§^ | N/A ^§^ | 2.80  (0.59-13.27) | 0.20 | N/A ^§^ | N/A ^§^ |
|  |  | Osteoporosis | N/A ^§^ | N/A ^§^ | 1.85  (0.37-9.33) | 0.46 | N/A ^§^ | N/A ^§^ | 0.43  (0.06-2.97 | 0.39 | 0.69  (0.18-2.66) | 0.59 | 1.65  (0.12-23.32) | 0.71 |
|  |  | Smoking | N/A ^§^ | N/A ^§^ | 0.79  (0.39-1.58) | 0.50 | 0.53  (0.18-1.63) | 0.27 | 0.80  (0.29-2.19) | 0.66 | 1.02  (0.53-1.98) | 0.95 | 19.89  (0.28-1397.17) | 0.17 |

^†^ Logistic regression analysis was performed in only maxillary posterior region.

^‡^ Total GBR means all types of hard tissue augmentation, including horizontal GBR, vertical GBR, or sinus floor elevation.

^§^ Estimates are not reliable because there were too few observations.

^¶^ Estimates are not reliable because there were no cases in the group
